# A Putative Alzheimer's Disease Risk Allele in *PCK1* Influences Brain Atrophy in Multiple Sclerosis

**DOI:** 10.1371/journal.pone.0014169

**Published:** 2010-11-30

**Authors:** Zongqi Xia, Lori B. Chibnik, Bonnie I. Glanz, Maria Liguori, Joshua M. Shulman, Dong Tran, Samia J. Khoury, Tanuja Chitnis, Todd Holyoak, Howard L. Weiner, Charles R. G. Guttmann, Philip L. De Jager

**Affiliations:** 1 Program in Translational NeuroPsychiatric Genomics, Department of Neurology, Brigham and Women's Hospital, Boston, Massachusetts, United States of America; 2 Program in Medical and Population Genetics, Broad Institute, Cambridge, Massachusetts, United States of America; 3 Department of Neurology, Brigham and Women's Hospital, Boston, Massachusetts, United States of America; 4 Center for Neurological Imaging, Department of Radiology, Brigham and Women's Hospital, Boston, Massachusetts, United States of America; 5 Center for Neurologic Diseases, Department of Neurology, Brigham and Women's Hospital, Boston, Massachusetts, United States of America; 6 Department of Biochemistry and Molecular Biology, University of Kansas Medical Center, Kansas City, Kansas, United States of America; 7 Institute of Neurological Sciences, National Research Council, Mangone, Italy; Julius-Maximilians-Universität Würzburg, Germany

## Abstract

**Background:**

Brain atrophy and cognitive dysfunction are neurodegenerative features of Multiple Sclerosis (MS). We used a candidate gene approach to address whether genetic variants implicated in susceptibility to late onset Alzheimer's Disease (AD) influence brain volume and cognition in MS patients.

**Methods/Principal Findings:**

MS subjects were genotyped for five single nucleotide polymorphisms (SNPs) associated with susceptibility to AD: *PICALM, CR1, CLU, PCK1*, and *ZNF224*. We assessed brain volume using Brain Parenchymal Fraction (BPF) measurements obtained from Magnetic Resonance Imaging (MRI) data and cognitive function using the Symbol Digit Modalities Test (SDMT). Genotypes were correlated with cross-sectional BPF and SDMT scores using linear regression after adjusting for sex, age at symptom onset, and disease duration. 722 MS patients with a mean (±SD) age at enrollment of 41 (±10) years were followed for 44 (±28) months. The AD risk-associated allele of a non-synonymous SNP in the *PCK1* locus (rs8192708^G^) is associated with a smaller average brain volume (*P* = 0.0047) at the baseline MRI, but it does not impact our baseline estimate of cognition. *PCK1* is additionally associated with higher baseline T2-hyperintense lesion volume (*P = *0.0088). Finally, we provide technical validation of our observation in a subset of 641 subjects that have more than one MRI study, demonstrating the same association between *PCK1* and smaller average brain volume (*P* = 0.0089) at the last MRI visit.

**Conclusion/Significance:**

Our study provides suggestive evidence for greater brain atrophy in MS patients bearing the *PCK1* allele associated with AD-susceptibility, yielding new insights into potentially shared neurodegenerative process between MS and late onset AD.

## Introduction

Multiple sclerosis (MS) is an inflammatory demyelinating disease of the central nervous system (CNS) that leads to brain atrophy and cognitive impairment [1], [2]. Cognitive dysfunction occurs in all stages of the disease, including the clinically isolated demyelinating syndrome (CIS) stage [3]. Impaired cognitive domains in MS include attention, processing speed, memory, executive function, and visual spatial perception [2], [4]. In Relapsing Remitting MS (RRMS), cortical demyelinating lesion burden and brain atrophy are both associated with cognitive dysfunction [5]. Similar to Alzheimer's disease (AD), brain atrophy and cognitive decline in MS occur in a progressive and non-remitting pattern but at a slower rate than AD for most MS patients [2], [4], [6].

Evidence supporting the existence of genetic factors that may influence brain atrophy and cognitive dysfunction in MS are emerging. For example, there is evidence that HLA DRB1*1501 and HLA B*4402 affect both brain atrophy and cognitive decline [7], [8], [9]. In this study, we explored the hypothesis that elements of the neurodegenerative process may be shared between MS and late onset AD, the most common neurodegenerative disease with manifestation of cognitive dysfunction typically after 65 years of age. The best-studied candidate gene for neurodegeneration in MS is *APOE* (apoliporotein E) with its ε4 allele conferring increased risk for AD, but the role of the *APOE* locus in MS remains unclear [10], [11], [12].

To address our hypothesis, we took a candidate gene approach and explored whether susceptibility alleles for AD other than *APOE* impact brain volume and cognition in a prospective cohort of MS patients. First, we selected three loci with genome-wide significant evidence of association with AD susceptibility as well as independent replication: *PICALM* (phosphatidylinositol-binding clathrin assembly protein) [13], *CR1* (complement component 3b/4b receptor 1) [14], and *CLU* (clusterin or apolipoprotein J) [13], [14]. We additionally selected *PCK1* (phosphoenolpyruvate carboxykinase 1) [15] and *ZNF224* (zinc finger protein 224) [16], two loci with suggestive evidence of association from AD genome-wide scans, and for which our group recently demonstrated association with age-related cognitive decline [17]. We present evidence that at least one of these loci may have a role in neurodegenerative events that occur in MS.

## Methods

### Participants

Using documents and protocols approved by the Institutional Review Board of Partners Healthcare, each subject gave written informed consent for their DNA, imaging data and clinical information for analysis. Subjects were enrolled between 2000 and 2010 at the Partners Multiple Sclerosis Center, and included participants in the Comprehensive Longitudinal Investigation of Multiple Sclerosis at the Brigham and Women's Hospital (CLIMB), an ongoing prospective cohort study based in the New England area. For this study, we only included subjects of self-reported non-Hispanic Caucasians between 18 and 65 years of age during the follow-up period who met the revised McDonald diagnostic criteria [18], [19] or had a diagnosis of CIS. Primary Progressive MS (n = 1) was excluded because of its possibly different pathophysiology from the other subsets of MS subjects and the fact that only one subject in this category had both genotype and imaging data. We limited analysis to three MS subcategories: relapsing-remitting (RRMS), secondary progressive (SPMS), progressive relapsing (PRMS).

### Genotyping Data

We considered five AD-associated SNPs in our cohort: *PICALM* (rs3851179), *CR1* (rs6656401), *CLU* (rs11136000), *PCK1* (rs8192708), and *ZNF224* (rs3746319). Genotyping were performed on the Affymetrix Genome-wide Human SNP Array 6.0 (Genechip 6.0) at the Broad Institute's Center for Genotyping and processed for quality control using the PLINK software suite as previously described [20], [21]. In short, we applied the standard quality control pipeline for subjects (genotype success rate >95%, genotype-derived gender concordant with reported gender, excess inter/intra-heterozygosity) and for SNPs (Hardy-Weinberg Equilibrium p>1×10^−6^; Minor Allele Frequency >0.01, genotype call rate >0.95).

### Assessment of HLA B*44 Status

Because the best surrogate marker (rs2743951) for HLA B*4402 was not genotyped in the array, a surrogate SNP (rs2523393) was selected based on its strong linkage disequilibrium (r^2^ = 0.92) with rs2743951 in the HapMAP CEU samples [21]. We classified HLA B*4402 based on the presence of the protective allele at this SNP (rs2523393^GG or GA^, 67% of the cohort) or the absence of the protective allele (rs2523393^AA^, 33% of the cohort).

### Clinical Data

Clinical data were obtained from the Partners MS Center Oracle relational database. At each clinic visit, the Extended Disability Status Scale (EDSS) score was measured on each patient. Disease duration was defined as the time interval from the self-reported symptom onset to recorded clinic visit.

### Radiographic Data

The neuroimaging approach in this cohort have been described in detail elsewhere [22]. In short, routine clinical MRI scans were obtained on 1.5 Tesla system (Signa, GE Medical Systems, Milwaukee, WI) using a standard bird-cage quadrature coil [23]. Images included T1-weighted proton density and T2-weighted axial images of 3-mm thick sections. Each brain was segmented using an automated template-driven pipeline, and brain parenchymal fraction (BPF) and T2-hyperintense lesion volume (T2LV) were calculated for each MRI scan [24], [25]. A detailed description of radiological analysis is described in the supplemental methods (Appendix S1).

### Cognitive Data

The Symbol Digit Modalities Test (SDMT) measures working memory and information processing speed using a complex visual scanning and tracking task. SDMT has been validated in MS and performance on SDMT is independent of physical disability [26], [27]. Subjects were given 90 seconds to substitute numbers for symbols as part of a pre-defined code with the final score being the total number of correct items.

### Statistical Methods

Demographic characteristics of the cohort are described using means and standard deviations for continuous variables and frequency and proportions for categorical variables. Linear regression was used to assess the relationship between each SNP and the primary outcomes (BPF and SDMT) at baseline (defined as study entry). For primary analyses, we implemented an additive model that examines the effect of each additional risk allele on outcome. We used linear mixed effects models to compare the rate of change over time in primary outcomes (BPF and SDMT) for the genotypes. If a significant association was found at *P*<0.005 (given the five SNPs and two primary outcomes), we examined the relationship further using (1) a dominant model comparing the homozygote for the non-risk allele to the combined heterozygote and homozygote for risk allele, and (2) a recessive model comparing the combined homozygote and heterozygote for the non-risk allele to the homozygote for the risk allele. For technical validation of significant association, we applied the same additive model and linear regression to assess the effect of genotype on primary outcomes at the last visit. All models were adjusted for sex, age of symptom onset, and disease duration.

In secondary analysis, we assessed whether HLA B*4402 interacts with the correlation between AD-associated SNP and MS outcome. If an interaction was found, subjects were stratified into two subgroups: HLA B*4402 positive (rs2523393^GG or GA^) or negative (rs2523393^AA^). If no interaction was found, we assessed HLA B*4402 status as a possible confounder covariate.

In addition to assessing each individual SNP, we calculated for each subject a cumulative genetic risk score (GRS), defined as the number of AD-associated risk alleles from our list of five candidate genes. The same linear regression model was used to examine the relationship between GRS and the outcomes.

Finally, if there is a significant association between a candidate SNP and BPF, we performed causal pathway analyses using Cooper's Local Causal Discovery algorithm [28] to determine whether the relationships between candidate SNP and BPF is mediated by T2LV. All tests were performed using SAS (version 9.2; SAS Institute, Cary, NC).

## Results

The characteristics of the 722 MS subjects of self-reported European ancestry with genotype and at least one MRI scan are summarized in **Table S1**. Of those, 318 subjects additionally have cognitive data. Serial MRI measures are available in 641 subjects. For the primary analysis, we used an additive model to assess whether the following late onset AD-associated variants influence brain atrophy and cognitive dysfunction in MS patients: *PICALM* (rs3851179), *CR1* (rs6656401), *CLU* (rs11136000), *PCK1* (rs8192708), and *ZNF224* (rs3746319). Genotype distributions for each SNP within our cohort are described in the supplemental section (**Table S2**).

### Genotype Correlation with Brain Volume and Cognition

**Figure 1:**
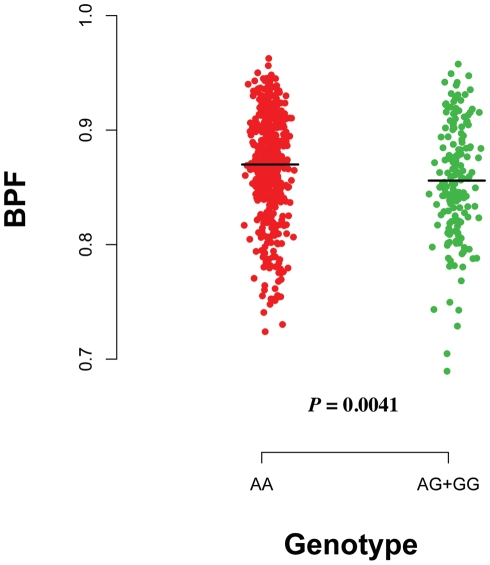
Effect of *PCK1* (rs8192708^G^) on the baseline brain volume using a dominant model. The dominant model provides the best fit for the effect of *PCK1* (rs8192708^G^) on the baseline brain volume as measured by brain parenchymal fraction (BPF). We compare the homozygotes for the non-risk allele (AA) to the combined heterozygotes (AG) and homozygotes (GG) for the risk allele. The minor allele (G) frequency is 0.14.

Of the tested AD-associated variants, the *CR1*, *CLU*, *PICALM* and *ZNF224* loci do not significantly impact baseline brain volume or cognition (**Table 1**). However, we found a significant association between rs8192708 in the *PCK1* locus and baseline brain volume (**Table 1**). Specifically, we examined whole brain volume using brain parenchymal fraction (BPF), and found that the average baseline BPF is smaller for each additional risk allele of *PCK1* (rs8192708^G^, beta = −0.0087, *P = *0.0047) (**Figure S1**). This result meets a Bonferroni-corrected threshold of significance (*P<*0.01) given five SNPs were tested with the primary outcome (BPF) using an additive model in the primary analysis. The effect of this risk allele may be dominant as *P-*value is 0.0041 in this model for baseline BPF (**Figure 1**). No significant association was observed when applying a recessive model (*P* = 0.37). The *PCK1* risk allele (rs8192708^G^) does not impact our baseline cognitive measure, SDMT, which is available in a subset of 318 subjects (beta = −1.23, *P = *0.36). Interestingly, the *PCK1* risk allele is correlated with greater average baseline T2-hyperintense lesion volume (beta = 0.83, *P = *0.0088). Finally, we did not observe a correlation between the *PCK1* risk allele and the baseline EDSS (beta = 0.20, *P* = 0.13).

**Table 1:** Genotype correlation with baseline outcomes at study enrollment.

				BRAIN VOLUME			COGNITION		
BASELINE					*BPF*			*SDMT*	
	SNP	Chr	Risk Allele	Beta	SE	*P*	Beta	SE	*P*
***PICALM***	rs3851179	11	C	−0.0017	0.0022	0.44	1.96	0.98	0.047
***CR1***	rs6656401	1	A	−0.0020	0.0026	0.44	−0.90	1.20	0.46
***CLU***	rs11136000	8	C	−0.0011	0.0021	0.60	−0.09	0.90	0.92
***PCK1***	rs8192708	20	G	−0.0087	0.0031	**0.0047**	−1.23	1.35	0.36
***ZNF224***	rs3746319	19	A	−0.0008	0.0028	0.77	2.16	1.19	0.07

Abbreviation: SNP, single nucleotide polymorphism; Chr, chromosome number; BPF, brain parechymal fraction; SDMT, symbol digit modalities test; *PICALM,* phosphatidylinositol-binding clathrin assembly protein; *CR1,* complement component 3b/4b receptor 1; *CLU,* clusterin or apolipoprotein J; *PCK1,* phosphoenolpyruvate carboxykinase 1; *ZNF224*, zinc finger protein.

Since an independent MS cohort of equivalent size with MRI data was not available for replication, we assessed the correlation between *PCK1* and BPF within our subjects at a different time point. Thus, we examined the subgroup of 641 subjects with two or more MRI scans (90% of the cohort) and found the same significant correlation between the rs8192708^G^ risk allele and smaller average BPF at the last MRI time point (beta = −0.009, *P = *0.0089). This provides a technical validation of our observation since the analysis uses an independent measure of BPF in our subjects.

### HLA B*44 and *PCK1*

In a secondary analysis, we investigated whether a surrogate marker for HLA B*4402 modifies the effects of the *PCK1* variant (rs8192708) on baseline brain volume given that we have previously reported an association between HLA B*4402 and higher BPF in MS subjects [7]. In our subjects, we found an interaction between HLA B*4402 and rs8192708 for BPF (*P* = 0.0095). In stratified analyses, the association between the rs8192708^G^ allele and smaller baseline BPF is driven by the HLA B*4402 positive subgroup (n = 463, beta = −0.014, *P* = 9.2×10^−5^). The HLA B*4402 negative subgroup demonstrates no significant association with the *PCK1* locus (n = 259, *P* = 0.53).

### Genetic Risk Score Correlation with Brain Volume and Cognition

**Figure 2:**
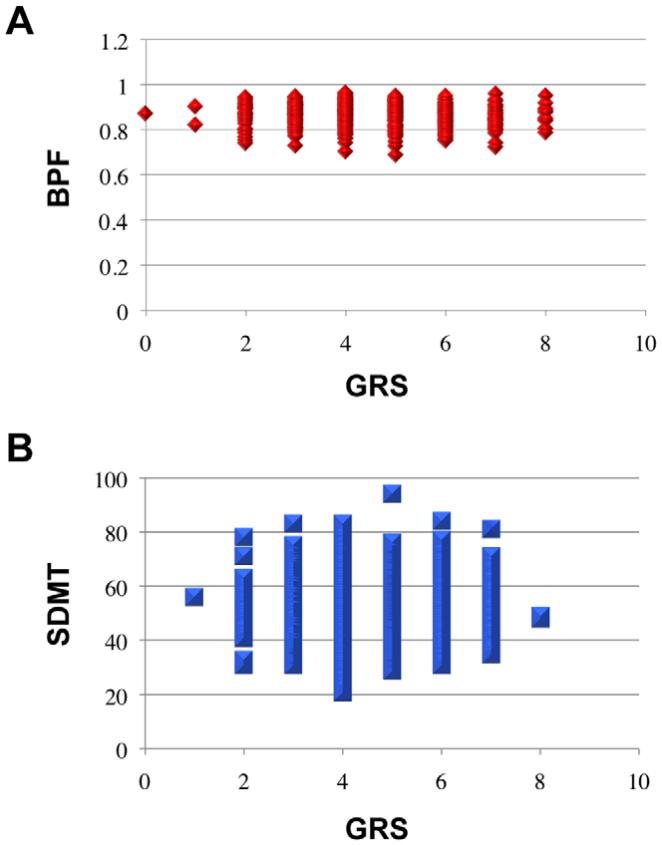
Genetic risk score and baseline outcome. Genetic risk score (GRS) for AD is calculated based on the number of AD-associated risk alleles at the five tested loci borne by each subject. GRS is not correlated with either (**A**) baseline brain parenchymal fraction (BPF, n = 722, *P* = 0.096) or (**B**) baseline cognition (SDMT, n = 318, *P* = 0.20).

To assess whether the five tested loci have a modest effect on our outcome measures, we calculated an aggregate, non-weighted genetic risk score (GRS) for each subject based on the number of AD-associated risk alleles at the five tested loci (range 0–10). When correlating GRS with our primary outcomes, we did not observe any statistically significant association with either brain volume or cognition at baseline (**Figure 2**).

### Causal Pathway Analysis

BPF is a measure of the neurodegenerative process, whereas T2LV provides a coarse estimate for the cumulative burden of inflammatory events in MS patients. Given current evidence suggesting that genetic susceptibility for MS and early events are largely related to immunologic dysfunction [21], we tested a model in which the effect of the *PCK1* allele, which is associated with both smaller BPF and greater T2LV burden, might follow a causal pathway such that inflammation triggers neurodegeneration. Specifically, we examined whether the effect of the rs8192708^G^ allele on T2LV mediates its effect on BPF by incorporating T2LV as a covariate in the model assessing BPF. With the T2LV covariate, the magnitude of the effect of rs8192708^G^ on BPF is reduced by 34% (beta = −0.0057±0.003, *P* = 0.048) but the effect is still present. Thus, the effect of the *PCK1* locus on BPF may be mediated in part by its effect on the extent of inflammatory disease as estimated by T2LV, but the residual association in the presence of the T2LV covariate suggests the existence of other mechanisms responsible for the effect of the *PCK1* locus on BPF.

## Discussion

In this study, we used a candidate gene approach to explore whether certain non-*APOE* genetic variants associated with AD susceptibility influence neurodegeneration in MS as assessed by measures of whole brain volume and cognition. Our principal finding is that among the 722 MS subjects with MRI data, those bearing the *PCK1* allele associated with AD susceptibility may have greater or accelerated brain atrophy. Specifically, an allele in the *PCK1* locus (rs8192708^G^) exhibits correlation with a smaller average BPF in our cohort of MS patients whose mean age at the time of imaging is at least 25 years younger than the mean age in late onset AD (75 years) [29]. This observation is intriguing and suggests that elements of the neurodegenerative process in MS and AD may be shared. Although the inciting events and certain key pathologic findings are clearly different between MS and AD, neuronal responses to perturbation in CNS parenchymal homeostasis may well be similar.

Given our sample size, we have some statistical power to discover modest effects such as the one we report in the *PCK1* locus but do not have sufficient power to exclude the involvement of the other four loci in MS-related neurodegeneration. Using the approach of GRS to aggregate the effects of candidate loci, our dataset does not exhibit evidence for modest effects in *PICALM, CR1, CLU* or *ZNF224*. Additional fine mapping will be required to determine whether rs8192708 is in fact the causal variant and whether *PCK1* is the causal gene for the observed association with brain atrophy in MS. We did not have access to another MS cohort of equivalent or greater sample size with MRI data to independently validate our observation, but such an effort is necessary in the future. Nonetheless, we provide evidence for technical validation of the association between *PCK1* and BPF by reproducing the result when analyzing the BPF measure from the last visit in the subset of subjects with more than one MRI scan.

**Figure 3:**
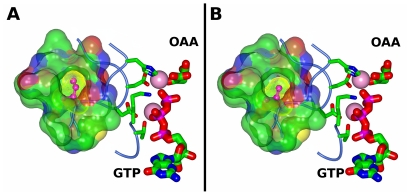
Structural models of cytosolic PEPCK. Based on previously published structural models of (**A**) rat cytosolic PEPCK (isoleucine 267) (PDBID 3DT7 [33]) and (**B**) human cytosolic PEPCK variant rs8192708^G^ (valine 267) (PDBID 1KHB [31]), the hydrophobic pocket to which the residue at position 267 belongs is rendered as a semi-transparent molecular surface colored by atom type (carbon, green; oxygen, red; nitrogen, blue; sulfur, yellow). The van der Waals surface for isoleucine (**A**) and valine (**B**) is rendered as a dotted yellow surface. The metal ions are rendered as pink spheres, the catalytic and metal binding residues are rendered as thin cylinders colored by atom type and the substrates oxaloacetate (OAA) and guanosine triphosphate (GTP) are rendered as thick sticks, colored by atom type and labeled.

*PCK1* encodes phosphoenolpyruvate carboxykinase 1 (PEPCK), a key enzyme in gluconeogenesis, responsible for converting oxaloacetate to phosphoenolpyruvate. PEPCK has a wide tissue expression pattern, including in the brain [30]. The AD risk-associated allele in *PCK1* (rs8192708^G^) causes a missense mutation (A to G) in the coding region, resulting in an isoleucine to valine substitution at residue 267 (I267V). The crystal structure and the kinetic properties of the rs8192708^G^ variant (V267) in human cytosolic PEPCK are available [31], [32], but *in vitro* data on the isoleucine variant (I267) in human cytosolic PEPCK has not been described. Comparison of the structural data on the rs8192708^G^ variant (V267) and I267 from the highly homologous rat cytosolic isozyme [33] (with 90% identity) illustrates the contribution of residue 267 to the formation of a hydrophobic pocket adjacent to the active site of the enzyme (**Figure 3**). Differences in the van der Waals surface for isoleucine and valine demonstrates that replacement of I267 by V267 creates a void in this pocket. However, the structural data suggest that the protein fold accommodates this void and that the I267V substitution does not lead to perturbation of the local structure. Furthermore, existing *in vitro* protein activity data on rat [34], [35] and human [32] PEPCK variants suggest that the I267V substitution does not have significant functional consequences in *in vitro* systems. It remains a possibility that the I267V substitution affects the stability and folding properties of PEPCK because of the reduced packing interaction (illustrated in **Figure 3**). Further *in vitro* investigations of the I267V substitution within a single isozyme are necessary to determine its structural and functional consequences.

Other variants in the *PCK1* locus have shown modest evidence of association with Type II Diabetes Mellitus (T2DM) [36], [37]. T2DM not only increases the risk for cerebral infarcts but also brain atrophy [38], and contributes to both vascular dementia and Alzheimer's disease [29]. We do not have T2DM as a systematic outcome in our MS subjects, but an intriguing question is whether the association of *PCK1* with greater T2LV may represent: (a) microvascular disease of the CNS parenchyma manifesting as white matter hyperintensities; (b) an increased burden of inflammatory disease activity; or (c) both. Regardless of the mechanism by which *PCK1* affects T2LV, this effect appears to partially mediate the effect of *PCK1* on brain atrophy, but much of the effect appears to be mediated by other mechanisms. Interestingly, this possible connection with an inflammatory effect is further elaborated in that the effect of the *PCK1* locus appears to be driven by those subjects (n = 463) who are positive for HLA B*4402, an MHC class I allele previously associated with larger BPF and lower T2LV in MS subjects [7]. Further investigation will be needed to validate the interaction between *PCK1* and HLA B*4402. Other MHC alleles, such as HLA DRB1*1501, are also associated with these outcomes [8], [9]. However, we have not reproduced the HLA DRB1*1501 association in our subjects and therefore did not pursue additional stratified analyses.

Our study does not address how the *PCK1* risk allele affect brain volume in healthy control subjects of the same age group since we do not have access to such a large cohort of subjects with genotype and MRI or cognitive measures. Evaluating the effect of the *PCK1* risk allele on the brain volume in healthy control subjects is an important question for future investigation.

Since we have longitudinal MRI data on 641 subjects, we also performed secondary analyses correlating the *PCK1* locus with the rate of change in BPF but did not find a significant association (*P* = 0.70). This is not surprising as the effect of *PCK1* on cross-sectional BPF and the average duration of follow-up (43.8 months) are both modest. The change in BPF annually or over the study period is too small when compared to the expected fluctuation in MRI measurement from test to test. Thus, our statistical power is limited in addressing this question, and we cannot yet distinguish whether the effect of the *PCK1* locus is one of accelerated brain atrophy in the context of MS or whether *PCK1* affects the maximal BPF attained in the course of brain development.

Similarly, we are limited in our analysis of cognitive function both because: (a) only a subset of subjects have cognitive outcome (n = 318, 44% of the cohort), and (b) although SDMT has become increasingly adopted as a measure of cognition in MS patients, it does not evaluate all cognitive domains and might thus underestimate the extent of cognitive dysfunction. Therefore, we cannot exclude the possibility that *PCK1* may influence cognitive manifestations of brain atrophy in MS.

In conclusion, our study suggests the existence of a shared process of neurodegeneration or response to neurodegeneration between MS and AD. Further work is needed to validate our findings and explore the impact of this *PCK1* variant on the structural alterations in the PEPCK protein.

## Supporting Information

Appendix S1 Supplemental Methods. Detailed Radiological Analysis.
(0.03 MB DOC)

Table S1 Demographics of the study population.
(0.06 MB DOCX)

Table S2 Genotype distribution of candidate genes within the cohort.
(0.05 MB DOC)

Figure S1 Effect of PCK1 (rs8192708^G^) on the baseline BPF using the additive model. The additive model demonstrates the effect of PCK1 (rs8192708^G^) on the baseline brain volume as measured by BPF. We compare the homozygotes for the non-risk allele (AA), heterozygotes (AG), and homozygotes (GG) for the risk allele. The minor allele (G) frequency is 0.14.
(9.12 MB TIF)
