# Supplementary material for: A Putative Alzheimer's Disease Risk Allele in PCK1 Influences Brain Atrophy in Multiple Sclerosis
Source: PLoS One. 2010 Nov 30;5(11):e14169. doi: 10.1371/journal.pone.0014169 (PMC2994939; doi:10.1371/journal.pone.0014169)
Supplement: Appendix S1 — Supplemental Methods. Detailed Radiological Analysis. (0.03 MB DOC) [file pone.0014169.s001.doc]

**APPENDIX S1: SUPPLEMENTAL METHODS**

**Detailed Radiological Analysis.**

Routine clinical magnetic resonance imaging (MRI) scans were obtained on 1.5 Tesla MR system (Signa, GE Medical Systems, Milwaukee, Wisconsin) using a standard bird-cage quadrature coil as part of routine clinical practice at the Partners MS Center. Images included dual-echo (proton density and T2-weighted) axial images (3-mm tick sections). Each brain is segmented using an automated template-driven segmentation pipeline termed TDS+ (24) by which each pixel within the intra-cranial cavity (ICC) is assigned to a tissue class including gray matter (GM), normal-appearing white matter (WM), white matter signal abnormalities or T2-hyperintense lesion volume (T2LV), and cerebrospinal fluid (CSF) based on their signal intensity characteristics as well as their anatomical location. Brain Parenchymal Fraction (BPF) is calculated from these measures using the following formula: BPF = (GM+WM+T2LV) / (GM+WM+T2LV+CSF). Starting in 2005, a stringent work flow driven structured visual quality control check was implemented for all quantitative MRI data (23).
