# Supplementary material for: A Putative Alzheimer's Disease Risk Allele in PCK1 Influences Brain Atrophy in Multiple Sclerosis
Source: PLoS One. 2010 Nov 30;5(11):e14169. doi: 10.1371/journal.pone.0014169 (PMC2994939; doi:10.1371/journal.pone.0014169)
Supplement: Table S1 — Demographics of the study population. (0.06 MB DOCX) [file pone.0014169.s002.docx]

**Table S1. Demographics of the study population.**

| **Measure** | **Value** |
| --- | --- |
|  |  |
| Number of Patients with Genotyping and MRI data | 722 |
| Subgroup with >1 MRI scan | 641 |
| Subgroup with Cognitive data | 318 |
| Female (%) | 547 (76%) |
| Age: Mean Years (SD) |  |
| Age at First Symptom | 32.2 (8.8) |
| Age at MS Diagnosis | 35.3 (9.1) |
| Age at Study Enrollment | 40.8 (9.6) |
| Race and Ethnicity |  |
| Non-Hispanic, Caucasian | 722 (100%) |
| Follow-up Duration: Mean Months (SD) | 43.8 (27.9) |
| Number of Visits: Median (Min, Max) |  |
| MRI | 5 (1, 17) |
| Cognitive | 3 (1, 9) |
| Number of Outcome Measures |  |
| MRI | 4297 |
| Cognitive | 601 |
| Disease Category at the Last Visit: Number (%) |  |
| Relapsing Remitting | 599 (83%) |
| Secondary Progressive | 92 (13%) |
| Clinically Isolated Syndrome | 26 (4%) |
| Progressive Relapsing | 4 (1%) |
| Primary Progressive | 0 |
| At Study Entry (Baseline) |  |
| Disease Duration: Mean Years (SD) | 8.4 (8.2) |
| EDSS: Median (Min, Max) | 1.5 (1.0, 2.5) |
| T2LV: Mean (SD) | 4.5 (4.0) |
| BPF: Mean (SD) | 0.87 (0.05) |
| SDMT: Mean (SD) | 54.7 (11.7) |
| At Last Study Visit |  |
| Disease Duration: Mean years (SD) | 11.5 (8.1) |
| EDSS: Median (Min, Max) | 1.5 (1.0, 3.0) |
| T2LV: Mean (SD) | 4.7 (4.0) |
| BPF: Mean (SD) | 0.86 (0.05) |
| SDMT: Mean (SD) | 56.6 (12.1) |

Abbreviation: SNP, single nucleotide polymorphism; SD, standard deviation; EDSS: Expaned Disability Status Scale; T2LV, T2 demyelinating lesion volume; BPF, brain parechymal fraction; SDMT, symbol digit modalities test.
