# Supplementary material for: A Putative Alzheimer's Disease Risk Allele in PCK1 Influences Brain Atrophy in Multiple Sclerosis
Source: PLoS One. 2010 Nov 30;5(11):e14169. doi: 10.1371/journal.pone.0014169 (PMC2994939; doi:10.1371/journal.pone.0014169)
Supplement: Table S2 — Genotype distribution of candidate genes within the cohort. (0.05 MB DOC) [file pone.0014169.s003.doc]

**Table S2. Genotype distribution of candidate genes within the cohort.**

| **Gene** | **SNP** | **Risk Allele** | **Genotype** | **% Cohort** | **% Cohort with SDMT** |
| --- | --- | --- | --- | --- | --- |
|  |  |  |  |  |  |
| ***PICALM*** | rs3851179 | C | C C | 38.7% | 37% |
|  |  |  | C T | 47.2% | 50% |
|  |  |  | T T | 14.1% | 13% |
| ***CR1*** | rs6656401 | A | A A | 3.9% | 2% |
|  |  |  | A C | 30.2% | 32% |
|  |  |  | C C | 65.9% | 66% |
| ***CLU*** | rs11136000 | C | C C | 40.5% | 40% |
|  |  |  | C T | 46.0% | 44% |
|  |  |  | T T | 13.5% | 15% |
| ***PCK1*** | rs8192708 | G | A A | 75.6% | 76% |
|  |  |  | A G | 22.6% | 22% |
|  |  |  | G G | 1.8% | 2% |
| ***ZNF224*** | rs3746319 | A | A A | 3.0% | 3% |
|  |  |  | A G | 25.8% | 29% |
|  |  |  | G G | 71.2% | 68% |
|  |  |  |  |  |  |

Abbreviation: SNP, single nucleotide polymorphism; SDMT, symbol digit modalities test. *PICALM,* phosphatidylinositol-binding clathrin assembly protein; *CR1,* complement component 3b/4b receptor 1; *CLU,* clusterin or apolipoprotein J; *PCK1,* phosphoenolpyruvate carboxykinase 1; *ZNF224*, zinc finger protein 224.
