# Supplementary figures and images for: A Putative Alzheimer's Disease Risk Allele in PCK1 Influences Brain Atrophy in Multiple Sclerosis
Source: PLoS One. 2010 Nov 30;5(11):e14169. doi: 10.1371/journal.pone.0014169 (PMC2994939; doi:10.1371/journal.pone.0014169)

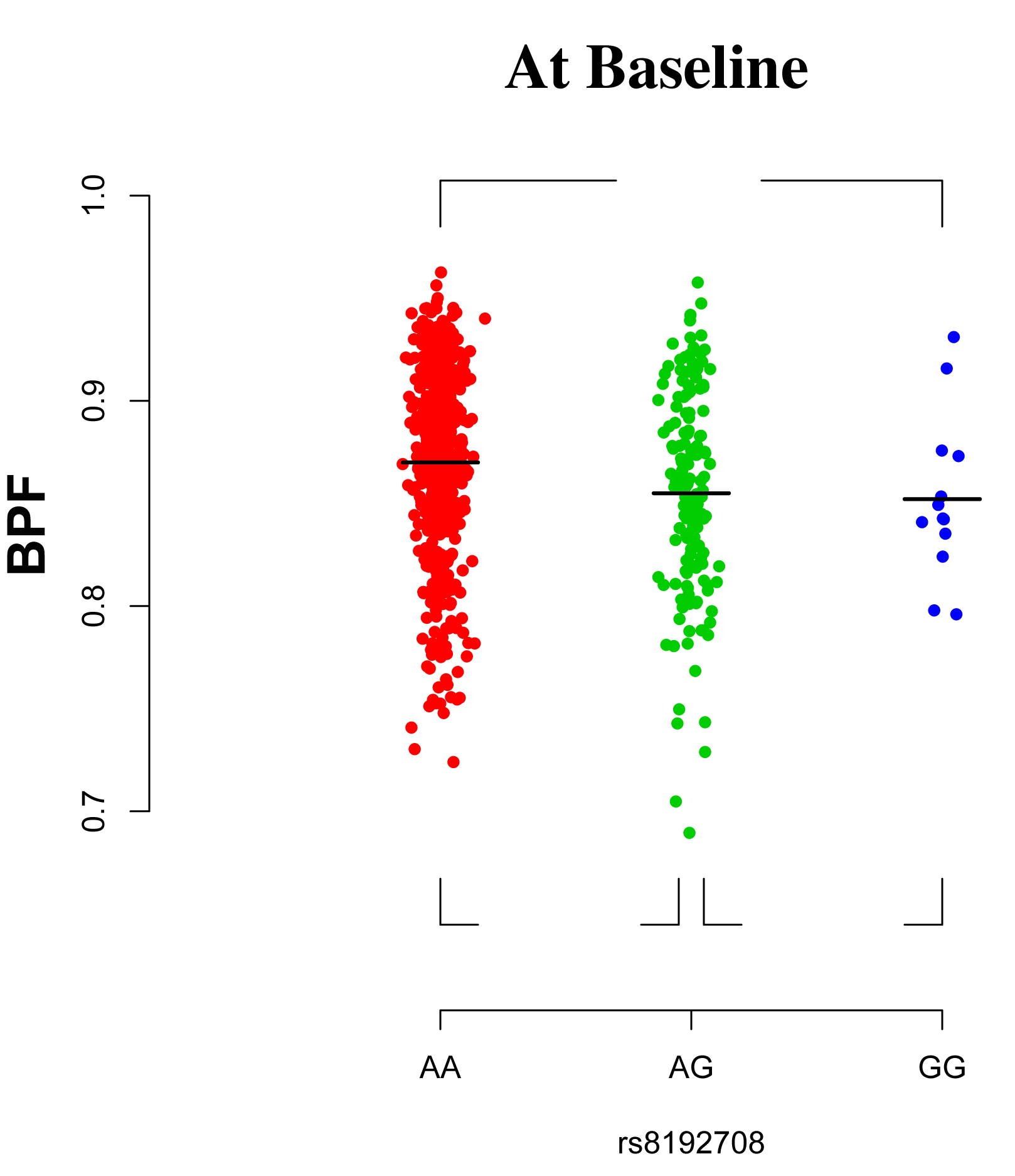

Supplement: Figure S1 — Effect of PCK1 (rs8192708G) on the baseline BPF using the additive model. The additive model demonstrates the effect of PCK1 (rs8192708G) on the baseline brain volume as measured by BPF. We compare the homozygotes for the non-risk allele (AA), heterozygotes (AG), and homozygotes (GG) for the risk allele. The minor allele (G) frequency is 0.14. (9.12 MB TIF) [file pone.0014169.s004.tif]
